# Hydrogen Peroxide Diffusion through Enamel and Dentin

**DOI:** 10.3390/ma11091694

**Published:** 2018-09-12

**Authors:** Carmen Llena, Oreto Martínez-Galdón, Leopoldo Forner, Lucía Gimeno-Mallench, Francisco J. Rodríguez-Lozano, Juan Gambini

**Affiliations:** 1 Department of Stomatology, Universitat de València, Valencia [Spain] C/ Gasco Oliag 1, 46010 Valencia, Spain; greti21@hotmail.com (O.M.-G.); forner@uv.es (L.F.); 2 Freshage Research Group, Department Physiology, Universitat de València, CIBERFES. INCLIVA. Avda. Blasco Ibáñez, 21, 46010 València, Spain; Lucia.gimeno@uv.es (L.G.-M.); Juan.Gambini@uv.es (J.G.); 3 School of Dentistry, Faculty of Medicine, University of Murcia, Avda. Marques de los Velez s/n, 30008 Murcia, Spain; fcojavier@um.es

**Keywords:** dental bleaching, carbamide peroxide, hydrogen peroxide, diffusion, light activation, application time

## Abstract

The purpose of this study was to evaluate the in vitro diffusion of commercial bleaching products (hydrogen peroxide (HP) or carbamide peroxide (CP) based) with different application protocols. Human enamel-dentin discs were obtained and divided into 20 groups. Four commercial products based on HP (Pola Office+(PO), Perfect Bleach (PB), Norblanc Office-automix (NO), and Boost (BT)), and one based on CP (PolaDay CP (PD)), were evaluated with different application protocols (3 applications × 10 min or 1 application × 30 min, with or without light activation). Artificial pulp chambers with 100 µL of a buffer solution were prepared. After each application, the buffer was removed and diffused HP was quantified by fluorimetry. Data were analyzed with two-way analysis of variance (ANOVA) and Tukey’s test. In groups where 3 × 10 min applications were done, after the first 10 min, PB, NO, and PD showed similar diffusion (*p* < 0.05). After the second and third applications, diffusion proved similar for PO and PD, while PB exhibited the greatest diffusion. In the 30 min application groups, PO and BT showed no significant differences (*p* > 0.05), with similar results for NO and PD. Comparing products with or without light activation, PO, BT, and PB showed significantly greater diffusion with light activation (*p* < 0.05). Reapplication, and light activation, increased HP diffusion independently of the concentration of the product.

## 1. Introduction

The use of hydrogen peroxide (HP) by itself, or released from other products, is currently the most widely used tooth bleaching technique. A number of factors intervene in HP diffusion through enamel and dentin, including the dental substrate itself, the concentration of the product, the application time, and the inclusion of some additional activation system [1,2,3]. Other factors, such as the viscosity of the gel, or certain substances, might also influence HP diffusion. The permeability of the mineralized dental tissues, and the low molecular weight of HP, allow the latter to diffuse more easily through the enamel and dentin [4].

Tooth bleaching occurs when reactive oxygen species (ROS) generated from HP react with organic chromophores through an oxidation process involving ionic exchange between the coloring agents and the ROS [5,6]. Micro-Raman spectroscopy has been used to demonstrate that HP penetrates the enamel without being consumed in the process, generating high free radical concentrations at the amelodentinal junction. Fourier transformation with photoacoustic infrared spectroscopy has shown that HP reaches the dentin and modifies its organic components. Consequently, HP diffusion dynamics are not a simple matter of penetration through dental structures, but are conditioned to the specificity of the dental tissue [7].

Maximum activation probably occurs where the organic component is greatest, in other words, in the vicinity of the amelodentinal junction [8]. Ubaldini et al. [7] found HP became consumed on penetrating through dentin—the proportion of HP remaining at the amelodentinal junction being about 63%, versus 37% after penetrating 3 mm into the dentinal layer.

Products used for application in dental offices contain concentrations of up to 40% in the case of HP, and above 16% in the case of carbamide peroxide (CP). The available data on diffusion dynamics are greater for HP-based products than for CP-based products.

Although the literature questions whether the use of bleaching agents with a high HP concentration is necessary, or even safe, such products are being used in single application, or with reapplications in the same clinical session, with the aim of incrementing the dental whitening effect [4,5,6]. Reapplication in the same session has been justified by the rapid degradation experienced by HP once applied [9]. However, some studies have shown the decomposition rate to be relatively low for the high concentration products used in dental offices [10,11].

With regard to the whitening effect, some authors have found bleaching to be faster with higher HP concentrations in the bleaching product [12,13], though other studies have obtained inconclusive results in this respect [14].

In relation to diffusion, a higher concentration of HP, and longer application times, have been associated with greater HP perfusion, and therefore with an increased risk of pulp damage [15].

Light activation of the products used in clinical practice is another source of controversy. While some authors describe greater bleaching action as a result of LED light application [16] without affecting the amount of HP reaching the pulp component [17], other investigators consider that light activation does not contribute to securing greater whitening, and moreover, increases the amount of HP that reaches the dental pulp [2].

With regard to the techniques used to measure HP diffusion, the most widely adopted strategy is that proposed by Mottola et al. in 1970 [18], based on spectrophotometry using leucocrystal violet and horseradish peroxidase. Soares et al. and Duque et al. [19,20] also employed leucocrystal violet and horseradish peroxidase combined with enzyme-linked immunosorbent assay (ELISA), while Yazaki et al. [21] used luminol chemiluminescence.

Fluorimetry is another more specific and sensitive technique for measuring HP levels [22]. This method involves measurement of the fluorescence emitted by the dimer formed between the diffused HP and homovanillic acid. The dimer is excited by a light beam at a wavelength of 312 nm, and emits fluorescence at a peak wavelength of 420 nm, which is detected by the fluorimeter. The dimerization reaction is mediated by peroxidase.

The null hypothesis was that peroxide diffusion will not be affected by (1) product, (2) application procedure, or (3) light activation.

The present in vitro study evaluates the diffusion capacity through 2 mm of enamel and 2 mm of dentin of bleaching products used in dental offices containing different concentrations of HP or CP, with different application times, and with or without light activation.

## 2. Materials and Methods

### 2.1. Sample Selection

This study was approved by the Ethics Committee of the University of Valencia (Valencia, Spain) (registry number: H1443515306255).

The study used fully impacted third molars that had been removed for therapeutic reasons from patients between 35 and 50 years of age, in order to secure a sample as homogeneous as possible, and involving teeth that had not been exposed to the oral environment, occlusal loading, or to other aggressive actions.

### 2.2. Sample Preservation and Preparation

Following extraction, the teeth were cleaned of organic remains and were checked for the absence of cracks, defects, or any changes in surface morphology, under ×10 magnification with a stereomicroscope (Stereo Discovery V8, Carl Zeiss Iberica S.L., Madrid, Spain). They were then immersed in 0.1% thymol solution at 4 °C for 48 h. After this period, the teeth were removed from the solution and immersed in distilled water at room temperature until use.

The samples were prepared by discarding the roots and sectioning the crowns mesiodistally using a low speed, water-cooled saw (Isomet, Buehler Ltd., Lake Bluff, IL, USA). Then the dentin layer was reduced by polishing with discs (Sof-Lex^TM^, 3M ESPE, St. Paul, MN, USA) to a thickness between 2 and 2.2 mm, measured with a digital caliper. The dentin surface was treated with ethylenediaminetetraacetic acid (EDTA) at a pH of 7.4 for 30 s for smear layer removal [19], and washed with deionized water. The enamel layer was not prepared.

The profile of each sample was traced over millimetered paper and scanned to calculate the surface in cm^2^ using the ImageJ program The area of the samples ranged from 0.4–0.6 cm^2^. Once prepared, the specimens were kept in individual containers in deionized water at 4 °C until use.

### 2.3. Preparation of the Experimental Reservoir

A heavy silicone (Aquasil Putty, Dentsply Sirona Iberia, York, PA, USA) reservoir was prepared with a capacity of 100 µL, simulating the pulp chamber. The sample was stabilized by producing a heavy silicone ring to which the specimen was anchored, with the dentin in contact with the buffer solution in the reservoir. The ring was perfectly adapted to the reservoir, and presented an upper window in which the study gel was deposited. Wax was placed to ensure sealing between the sample and the silicone ring, employing a procedure similar to that described by Torres et al. [23].

### 2.4. Products Used and Bleaching Protocol

After preparation and calculation of the surface area, the specimens were randomly divided into 20 groups (*n*_i_ = 5) according to the bleaching product used and the activation technique involved, and with or without light activation (Table 1). Four commercial HP products at a concentration of between 35% and 40%, and one product based on CP at a concentration of 35%, were used. In the groups subjected to three applications of 10 min each, with or without activation, the buffer solution in the reservoir was removed after each application, and the diffused HP was quantified. In the groups subjected to a single application, HP diffusion was measured after 30 min of application of the bleaching product.

After fitting the specimen in the anchoring ring with wax sealing to prevent the bleaching product from penetrating into the reservoir, 0.04 mL of the study gel was applied to each specimen with a spatula. The samples were covered with transparent film during the diffusion process.

Light activation of the corresponding samples was made using a Radii Cal LED lamp with a power of 1200 mW/cm^2^ (SDI, Bayswater, Victoria, Australia) at the distance of 1 mm from the sample for 3 min in each application

### 2.5. Measurement of HP Diffusion

Fluorimetry was used to evaluate HP diffusion with the different commercial products and application protocols. We first plotted a standard reference line by adding 2 μL of 33% HP (JT Baker Analyzed, Phillipsburg, NJ, USA) to an Eppendorf tube with 1998 μL of Milli-Q water to obtain a peroxide dilution of 1/1000. Precise quantification of the concentration of HP was carried out by measuring the absorbance at 420 nm, and applying the Lambert–Beer equation. Based on this known initial concentration, we performed serial dilutions to plot the line. A mix was prepared composed of 1955 µL of peroxide buffer, 20 µL of homovanillic acid, and 5 µL of peroxidase. The known concentrations of HP were added to the mix, followed two minutes later by glycine-EDTA buffer to stop the reaction. The samples were placed in the fluorimeter (Luminescence Spectrometer, LS 50 B, Perkin Elmer, Waltham, MA, USA) and subjected to excitation with a light source at a wavelength of 320 nm. Measurements were made of the emission of each dilution at 420 nm. This procedure yielded a standard reference line for extrapolation of the results of the study samples.

### 2.6. Statistical Analysis

Knowing the buffer volume, the area of the sample, and the application times of each product, we calculated the amount of HP diffused over time and per surface unit (nmol/cm^2^). The diffusion values per surface unit for each application time between the groups with and without light activation were compared using the Mann–Whitney U-test. Comparisons of the different application times within each group were made using two-way ANOVA and Tukey’s test. Statistical significance was considered for *p* < 0.05.

## 3. Results

With regard to the influence of the number of applications, the amount of diffused HP was seen to increase in each application for all the products tested. Likewise, after the third 10 min application, the amount of diffused HP was greater than after a single 30 min application (Table 2).

**Figure 1:**
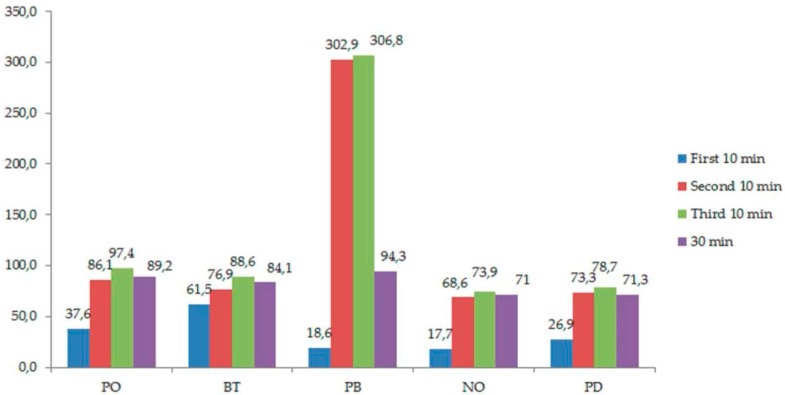
Mean diffusion values in each time without light activation.

After the first application of 10 min without activation, PB, NO, and PD showed similar diffusion without significant differences (*p* < 0.05), with BT showing the highest value (61.5 ± 6.1 nmol/cm^2^), followed by PO (37.6 ± 5 nmol/cm^2^). After the second and third application of 10 min, PO, BT, and PD showed similar diffusion values, and NO was the product with the smallest diffusion. In comparison, PB showed far higher diffusion values than the rest of the products in both applications (Table 2 and Figure 1). After 30 min of application, PO and BT showed similar behavior, with no significant differences between them (*p* > 0.05). In the same way, NO and PD also displayed similar behavior. The highest diffusion values corresponded to PB (Table 2 and Figure 1).

**Figure 2:**
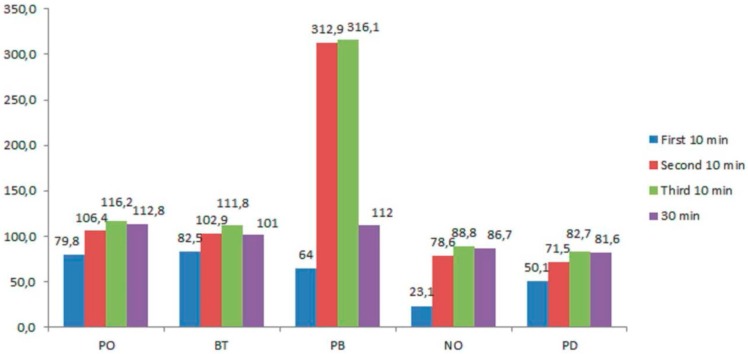
Mean diffusion values in each time with light activation.

In those groups in which light activation was used, PO and BT were seen to behave similarly at all application times, with no significant differences between them (*p* > 0.05). In the same way, NO and PD also displayed similar behavior. However, PB exhibited a pattern similar to that of the groups without light activation, showing a significant increase in diffusion (*p* < 0.05) versus the other groups from the second application (Table 2 and Figure 2).

On comparing each of the products with or without activation, PO and BT showed significantly greater diffusion with activation at all application times (*p* < 0.05). In contrast, while NO and PD showed increased diffusion when subjected to light activation, the differences were not statistically significant (*p* > 0.05). Lastly, PB showed significantly greater diffusion with light activation after 10 min and 30 min of application (Table 2).

## 4. Discussion

Since the dentin tubule diameter influences dentin permeability [24,25,26], and structural changes occur in the course of the life of the tooth, we decided to use fully impacted third molars removed for therapeutic reasons in our study, with the aim of obtaining as homogeneous a sample as possible.

Discs were sectioned presenting an enamel thickness of 2 mm and a dentin thickness between 2 and 2.2 mm [27], with an area of between 0.4 and 0.6 cm^2^. The central zones of the buccal and lingual surfaces of the molars were used for this purpose.

Fluorimetry is based on the absorption of radiation by the study product (in this case HP), and its subsequent emission. In the fluorescence phenomenon, a molecule absorbs electromagnetic radiation, and is transformed from the basal state to an activated state with the excitation of its electrons. The emitted radiation wavelength is different from the absorbed radiation wavelength, and this can be used to identify and quantify substances [22].

There is great heterogeneity among the different published studies in terms of the measurement units used to measure HP diffusion, for example, μg/mL [27], μg [28], or nmoles/min [29]. In contrast to most publications, in the present study we considered sample surface and calculated total HP diffusion in nmoles/cm^2^, with the purpose of taking into account the influence of sample size upon the results obtained.

Enamel and dentin behave as semipermeable membranes allowing the diffusion of HP according to Fick’s second law [30]. Most studies on peroxide diffusion through dental structures are consistent with this law [27,31].

The null hypothesis of this study was rejected, since the diffusion was affected by product, application procedure, and light activation. Nevertheless, the concentration of the product did not correlate with the amount of diffused peroxide.

Products containing a high concentration of HP or CP can only be used in a clinical setting. The application times recommended by the manufacturers vary from one product to another, though most recommend reapplications in the same clinical session. The findings of the present study show that reapplication results in greater HP diffusion than a single application independently of the product used, its HP concentration, or whether light activation is used or not. These observations are concordant with those of Soares et al. and de Almeida et al. [15,27]. Marson et al. [11] evaluated the decomposition of a commercial HP product applied for 45 min in contact with enamel, and found that only 11.1% of the HP had decomposed in that time, thus suggesting that reapplication is not necessary, particularly when considering that the amount of HP potentially capable of reaching the pulp tissue increases when reapplications are made. Accordingly, and as seen in our study, it would not be advisable from the clinical perspective to perform product reapplications in the same clinical session. Most authors report similar whitening effects with high concentration products following either a single application per session, or reapplications [14,27,32].

Light activation resulted in increased diffusion in all cases. Significantly greater diffusion was recorded for BT and PO in all applications, for PB after 10 min and 30 min applications, with no significant increments in the case of NO or PD. Although all the HP-based products used indicate that light activation is not necessary, most manufacturers mention that activation can be used. Although light activation is not recommended when using CP, we applied such activation in our study in order to evaluate its effect. Significant differences were observed only after a 10 min application. Studies that have evaluated diffusion time and the amount of diffused HP, with or without light activation, have revealed that the diffusion time decreases, and the amount of diffused HP increases, when a light source is used [27]. However, Kwon et al. reported that the amount of diffused HP was not affected by LED activation [33].

Light activation produces a degree of warming of the gel, causing molecular movement to increase, and the system to acquire greater kinetic energy. According to molecular collision theory, part of the kinetic energy is consumed when molecular collisions occur, with a weakening and disruption of the molecular bonds, and this in turn facilitates reactions. In this regard, on elevating the temperature, the gel’s molecular collisions become more frequent, thereby increasing the probability of reactions and accelerating the diffusion rate [34].

With respect to the concentration of the product, we found no correlation between the amount of diffused HP and the concentration of the gel applied. The products BT (40%) and PO (37.5%) showed very similar behavior in the course of the study. In contrast, PO and NO, which contain the same concentration (37.5%), exhibited significantly different diffusion levels in most experiments, the behavior of NO being very similar to that of PD (35% CP, equivalent to an HP concentration of about 12%). The behavior of PB (35% HP) was surprising, since with the exception of the first application of 10 min, its diffusion values were higher than those of PO and BT, and the diffusion dynamics of PB in the second and third reapplication were very different from those of the rest of the products. In this sense, neither the HP concentration (35%) or the pH of the product (7.3) would explain a behavior so different from that of the other products, leading us to believe that it contains other added components not identified that influence diffusion.

Diffusion capacity and the biological effects of different commercial bleaching products on human dental pulp stem cells (*h*DPSCs) has been evaluated. The findings were that different commercial products with similar HP concentrations exhibited different biological effects on *h*DPSCs [35] than similar concentrations of CP and HP.

The literature describes concentration as a determinant factor in HP diffusion, though some studies have used dilutions from pure HP, not commercial products [15,36]. On the other hand, a number of authors have found no significant differences in the amount of diffused HP according to the concentration of the product [37,38].

All the products evaluated in the present study were of neutral pH as stated by the manufacturers, though in the case of PB the stated pH was 7.3 at 20 °C. We did not take gel temperature into account in our series, and in this regard the pH may possibly differ as the temperature changes and influence the diffusion dynamics. In affect, at pH 9 dissociation is 2.7-fold greater than at pH 4.4; consequently, increased HP dissociation into free radicals within the dental structures means that less HP will reach the pulp tissues. The pH value is therefore an important factor in the diffusion of HP [37].

Other factors that might influence the diffusion capacity of HP are the product added and the viscosity of the gel, which could affect the wettability of the enamel surface [38,39]. In addition, it must be taken into account that the absence of pulpo-dentinal flux and pressure in in vitro studies could increase the diffusion when compared with the in vivo situation.

## 5. Conclusions

Based on the results obtained in this in vitro study, it can be concluded that a longer application time, reapplication, and light activation increases HP diffusion. The concentration of the product is not correlated to the amount of diffused HP.

## Figures and Tables

**Table 1:** Bleaching products evaluated and application procedure.

Product	Without Light Activation		With Light Activation	
Opalescence Boost, HP 40% Ultradent, South Jordan, UT, USA (BT)	3 × 10 min	1 × 30 min	3 × (3 min activ. + 5 min without activ.)	1 × (3 min activ. + 25 min without activ.)
NorBlanc Office Automix, HP 37.5%—Normon, Madrid, Spain (NO)	3 × 10 min	1 × 30 min	3 × (3 min activ. + 5 min without activ.)	1 × (3 min activ. + 25 min without activ.)
Pola Office +, HP 37.5%—SDI, Bayswater, Victoria, Australia (PO)	3 × 10 min	1 × 30 min	3 × (3 min activ. + 5 min without activ.)	1 × (3 min activ. + 25 min without activ.)
Perfect Bleach, HP 35%—Voco, Cuxhaven, Germany (PB)	3 × 10 min	1 × 30 min	3 × (3 min activ. + 5 min without activ.)	1 × (3 min activ. + 25 min without activ.)
PolaDay CP, CP 35%—SDI, Bayswater, Victoria, Australia (PD)	3 × 10 min	1 × 30 min	3 × (3 min activ. + 5 min without activ.)	1 × (3 min activ. + 25 min without activ.)

**Table 2:** Mean diffusion values (95% confidential interval) in nmols/cm^2^.

	Without Light Activation				
	Pola Office + (PO)	Boost (BT)	Perfect Bleach (PB)	NorBlanc Office Automix (NO)	PolaDay CP (PD)
First 10 min	37.6 (31.4–43.7)	61.5 (53.9–69)	18.6 (7.4–29.6) ^a^	17.7 (11.2–24.0) ^a^_1_	26.9 (19.3–34.5) ^a^
Second 10 min	86.1 (69.7–102.3) ^a^	76.9 (74.5–79.2) ^ab^	302.9 (264.9–340.9) _1_	68.6 (60.2–76.8) ^b^_2_	73.3 (64.3–82.1) ^a^_1_
Third 10 min	97.4 (76.5–118.2) ^a^	88.6 (80–97.1) ^a^	306.8 (259–354.6) _2_	73.9 (65.8–82) ^b^_3_	78.7 (67.3–90) ^b^_2_
30 min	89.2 (83.6–99.4) ^a^	84.1 (79.3–96.2) ^a^	94.3 (88.46–100)	71.0 (59.3–83.3) ^b^_4_	71.3 (58.3–84.6) ^b^_3_
	**With Light Activation**				
First 10 min	79.8 (163.2–96.4) ^b^	82.5 (63.9–100.9) ^b^	64 (52.1–75.9) ^a^	23.1 (11–35) _1_	50.1 (39.9–60.2) ^a^
Second 10 min	106.4 (93.3–119.4) ^b^	102.9 (187.7–1) ^b^	312.90 (281.4–344.2) _1_	78.6 (69.2–87.8) ^a^_2_	71.5 (65.6–77.5) ^a^_1_
Third 10 min	116.2 (102.2–130.1) ^b^	111.8 (97.7–125.9) ^b^	316.15 (286.8–345.3) _2_	88.8 (75–102.6) ^a^_3_	82.7 (78.9–86.4) ^a^_2_
30 min	112.8 (98.2–126.3) ^b^	101 (93.7–113.8) ^b^	112.01 (98.6–123.7) ^b^	86.7 (65.3–98.6) ^a^_4_	81.6 (69.4–96.7) ^a^_3_

The same superscript letter in each row expresses groups without significant differences; the same subscript number in each column expresses groups without significant differences.

## References

[1] Bowles W.H., Ugwuneri Z. (1987). Pulp chamber penetration by hydrogen peroxide following vital bleaching procedures. J. Endod..

[2] Camargo S.E., Cardoso P.E., Valera M.C., de Araújo M.A., Kojima A.N. (2009). Penetration of 35% hydrogen peroxide into the pulp chamber in bovine teeth after LED or Nd: YAG laser activation. Eur. J. Estht. Dent..

[3] Sulieman M. (2004). An overview of bleaching techniques: I. History, chemistry, safety and legal aspects. Dent. Updat..

[4] Alqahtani M.Q. (2014). Tooth-bleaching procedures and their controversial effects: A literature review. Saudi Dent. J..

[5] Joiner A. (2006). The bleaching of teeth: A review of the literature. J. Dent..

[6] Minoux M., Serfaty R. (2008). Vital tooth bleaching: Biologic adverse effects––A review. Quintessence Int..

[7] Ubaldini A.L., Baesso M.L., Medina-Neto A., Sato F., Bento A.C., Pascotto R.C. (2013). Hydrogen peroxide diffusion dynamics in dental tissues. J. Dent. Res..

[8] Xu B., Li Q., Wang Y. (2011). Effects of pH values of hydrogen peroxide bleaching agents on enamel surface properties. Oper. Dent..

[9] Al-Qunaian T.A., Matis B.A., Cochra M.A. (2003). In vivo kinetics of bleaching gel with three-percent hydrogen peroxide within the first hour. Oper. Dent..

[10] Matis B.A., Gaiao U., Blackman D., Schultz F.A., Eckert G.J. (1999). In vivo degradation of bleaching gel used in whitening teeth. J. Am. Dent. Assoc..

[11] Marson F.C., Gonçalves R.S., dos Santos P.H., Cintra L.T., Pascotto R.C., Santos P.H., Briso A.L. (2015). Penetration of hydrogen peroxide and degradation rate of different bleaching product. Oper. Dent..

[12] Sulieman M., Addy M., MacDonald E., Rees J.S. (2004). The effect of hydrogen peroxide concentration on the outcome of tooth whitening: An in vitro study. J. Dent..

[13] Da Costa J.B., McPharlin R., Paravina R.D., Ferracane J.L. (2010). Comparison of at-home and in-office tooth whitening using a novel shade guide. Oper. Dent..

[14] Matis B.A., Cochran M.A., Franco M., Al-Ammar W., Eckert G.J., Stropes M. (2007). Eight in-office tooth whitening systems evaluated in vivo: A pilot study. Oper. Dent..

[15] Soares D.G., Basso F.G., Pontes E.C., da FR Garcia L., Hebling J., de Souza Costa C.A. (2014). Effective tooth-bleaching protocols capable of reducing H(2)O(2) diffusion through enamel and dentine. J. Dent..

[16] Llena C., Forner L., Vazquez M. (2016). Hydrogen peroxide diffusion with and without light activation. Int. J. Esthet. Dent..

[17] Kwon S.R., Li Y., Oyoyo U., Aprecio R.M. (2012). Dynamic model of hydrogen peroxide diffusion kinetics into the pulp cavity. J. Contemp. Dent. Pract..

[18] Mottola H.A., Simpson B.E., Gorin G. (1970). Absorptiometric determination of hydrogen peroxide in submicrogram amounts with leuco crystal violet and peroxidase as catalyst. Anal. Chem..

[19] Soares D.G., Ribeiro A.P., da Silveira Vargas F., Hebling J., de Souza Costa C.A. (2013). Efficacy and cytotoxicity of a bleaching gel after short application times on dental enamel. Clin. Oral. Investig..

[20] Duque C.C., Soares D.G., Basso F.G., de Souza Costa C.A. (2014). Bleaching effectiveness, hydrogen peroxide diffusion, and cytotoxicity of a chemically activated bleaching gel. Clin. Oral. Investig..

[21] Yazaki K., Kawada E., Oda Y. (2003). An evaluation of the penetration of peroxide from tooth-whitener. Biomed. Res..

[22] Barja de Quiroga C. (1999). Mitochondrial oxygen radical generation and leak: Sites of produccion in states 4 and 3, organ specificity and relation to aging and longevity. J. Bioenerg. Biomembr..

[23] Torres C.R., Souza C.S., Borges A.G., Huhtala M.F., Caneppele T.M. (2013). Influence of concentration and activation on hydrogen peroxide diffusion through dental tissues in vitro. Sci. World J..

[24] Hairul Nizam B.R., Lim C.T., Chng H.K., Yap A.U.J. (2005). Nanoindentation study of human premolars subjected to bleaching agent. J. Biomech..

[25] Chng H.K., Ramli H.N., Yap A.U.J., Lim C.T. (2007). Effect of hydrogen peroxide on intertubular dentine. J. Dent..

[26] Zimmerman B., Datko L., Cupelli M., Alapati S., Dean D., Kennedy M. (2010). Alteration of dentin-enamel mechanical properties due to dental whitening treatments. J. Mech. Behav. Biomed. Mater..

[27] De Almeida L.C., Soares D.G., Gallinari M.O., de Souza Costa C.A., Dos Santos P.H., Briso A.L. (2005). Color alteration, hydrogen peroxide diffusion, and cytotoxicity caused by in-office bleaching protocols. Clin. Oral. Investig..

[28] Gökay O., Müjdeci A., Algn E. (2004). Peroxide penetration into the pulp from whitening strips. J. Endod..

[29] Hannig C., Weinhold H.C., Becker K., Attin T. (2011). Diffusion of peroxides through dentine in vitro with and without prior use of a desensitizing varnish. Clin. Oral. Investig..

[30] Kalia Y.N., Guy R.H. (2001). Modeling transdermal drug release. Adv. Drug. Deliv. Rev..

[31] Soares D.G., Marcomini N., Basso F.G., Pansani T.N., Hebling J., de Souza Costa C.A. (2016). Indirect cytocompatibility of a low-concentration hydrogen peroxide bleaching gel to odontoblast-like cells. Int. Endod. J..

[32] Dietschi D., Rossier S., Krejci I. (2006). In vitro colorimetric evaluation of the efficacy of various bleaching methods and products. Quintessence Int..

[33] Kwon S.R., Oyoyo U., Li Y. (2013). Effect of light activation on tooth whitening efficacy and hydrogen peroxide penetration: An in vitro study. J. Dent..

[34] Soares D.G., Basso F.G., Hebling J., de Souza Costa C.A. (2014). Concentrations of and application protocols for hydrogen peroxide bleaching gels: Effects on pulp cell viability and whitening efficacy. J. Dent..

[35] Llena C., Collado-González M., Tomás-Catalá C.J., García-Bernal D., Oñate-Sánchez R.E., Rodríguez-Lozano F.J., Forner L. (2018). Human Dental Pulp Stem Cells Exhibit Different Biological Behaviours in Response to Commercial Bleaching Products. Materials.

[36] Mena-Serrano A.P., Parreiras S.O., do Nascimento E.M., Berger S.B., Loguercio A.D., Reis A. (2015). Effects of the concentration and composition of in-office bleaching gels on hydrogen peroxide penetration into the pulp chamber. Oper. Dent..

[37] Berger S.B., Tabchoury C.P., Ambrosano G.M., Giannini M. (2013). Hydrogen peroxide penetration into the pulp chamber and dental permeability after bleaching. Gen. Dent..

[38] Buzoglu H.D., Gümüsderelioğlu M., Rotstein I. (2009). Effect of bleaching agents on surface free energy parameters of resin composite coated with saliva biofilm. Am. J. Dent..

[39] Kwon S.R., Pallavi F., Shi Y., Oyoyo U., Mohraz A., Li Y. (2018). Effect of bleaching gel viscosity on tooth
whitening efficacy and pulp chamber penetration: An in vitro study. Oper. Dent..

